# UFM1 suppresses invasive activities of gastric cancer cells by attenuating the expression of PDK1 through PI3K/AKT signaling

**DOI:** 10.1186/s13046-019-1416-4

**Published:** 2019-09-18

**Authors:** Jian-Xian Lin, Xin-Sheng Xie, Xiong-Feng Weng, Sheng-Liang Qiu, Changhwan Yoon, Ning-Zi Lian, Jian-Wei Xie, Jia-Bin Wang, Jun Lu, Qi-Yue Chen, Long-Long Cao, Mi Lin, Ru-Hong Tu, Ying-Hong Yang, Chang-Ming Huang, Chao-Hui Zheng, Ping Li

**Affiliations:** 1 Department of Gastric Surgery, Fujian Medical University Union Hospital, Fuzhou, 350001 Fujian Province China; 2 Key Laboratory of Ministry of Education of Gastrointestinal Cancer, Fujian Medical University, Fuzhou, 350108 Fujian Province China; 3 Fujian Key Laboratory of Tumor Microbiology, Fujian Medical University, Fuzhou, 350108 Fujian Province China; 4 Department of Pathology, Fujian Medical University Union Hospital, Fuzhou, 350001 Fujian Province China; 5 Department of Surgery, Memorial Sloan Kettering Cancer Center, New York, NY USA

**Keywords:** UFM1, PDK1, Gastric cancer, EMT

## Abstract

**Background:**

UFM1 has been found to be involved in the regulation of tumor development. This study aims to clarify the role and potential molecular mechanisms of UFM1 in the invasion and metastasis of gastric cancer.

**Methods:**

Expression of UFM1 in gastric tumor and paired adjacent noncancerous tissues from 437 patients was analyzed by Western blotting, immunohistochemistry, and realtime PCR. Its correlation with the clinicopathological characteristics and prognosis of gastric cancer patients was analyzed. The effects of UFM1 on the invasion and migration of gastric cancer cells were determined by the wound and trans-well assays, and the effect of UFM1 on subcutaneous tumor formation was verified in nude mice. The potential downstream targets of UFM1 and related molecular mechanisms were clarified by the human protein kinase assay and co-immunoprecipitation technique.

**Results:**

Compared with the corresponding adjacent tissues, the transcription level and protein expression level of UFM1 in gastric cancer tissues were significantly downregulated (*P* < 0.05). The 5-year survival rate of gastric cancer patients with low UFM1 expression was significantly lower than the patients with high UFM1 expression (42.1% vs 63.0%, *P* < 0.05). The invasion and migration abilities of gastric cancer cells with stable UFM1 overexpression were significantly decreased, and the gastric cancer cells with UFM1 stable knockdown showed the opposite results; similar results were also obtained in the nude mouse model. Further studies have revealed that UFM1 could increase the ubiquitination level of PDK1 and decrease the expression of PDK1 at protein level, thereby inhibiting the phosphorylation level of AKT at Ser473. Additionally, the effect of UFM1 on gastric cancer cell function is dependent on the expression of PDK1. The expression level of UFM1 can improve the poor prognosis of PDK1 in patients with gastric cancer.

**Conclusion:**

UFM1 suppresses the invasion and metastasis of gastric cancer by increasing the ubiquitination of PDK1 through negatively regulating PI3K/AKT signaling.

## Background

Gastric cancer is a malignant tumor with a high incidence and mortality. Currently, the overall therapeutic effect of gastric cancer treatment is not satisfactory, and the 5-year survival rate is still low [1, 2]. Recurrence and metastasis of gastric cancer is the main causes of death and also a complex pathological process caused by a series of molecular changes, while the clinical treatment of recurrence and metastasis is still not satisfactory [3]. Therefore, the study of key molecular events and signaling pathways in the development and metastasis of gastric cancer is helpful for revealing the mechanism of gastric carcinogenesis, development and improving the diagnosis of early gastric cancer, even providing great significance for the treatment of advanced gastric cancer.

UFM1 is a small molecule ubiquitin protein that was first discovered by Komatsu et al. in 2004 [4]. It consists of 85 amino acids, has a modification function similar to that of ubiquitin, which is covalently bound to other proteins such as ubiquitin molecules. This phenomenon is called UFM1 ufmylation [5]. UFM1 and its modification system are widely and conservatively presented in animals and plants, especially in cells that secrete proteins, which are mainly distributed in the cytoplasm and nucleus [6]. At present, UFM1 and its modification system are involved in a variety of pathophysiological processes, and participate in biological processes such as the cell cycle, cell survival, hypoxia tolerance, and fatty acid β oxidation [7–9]. UFM1 usually exists as the precursor form inside cells. For UFM1 to play its role in attaching to the target protein, it must be digested to expose the glycine at the carboxy terminus, followed by a series of enzyme cascades [10]. The enzyme cascades of the UFM1 system include the initial activation of the treated UFM1 by UBA5, followed by transfer to UFC1 and then to UFL1. UFL1 recognizes the target protein and helps UFM1 bind to the target protein. Finally, UFM1 processes the target protein to attain its important biological function [11]. As an important transduction molecule of PI3K signaling pathway, the binding of PDK1 to PIP3 plays an important role in the activation of AKT and other kinases. It regulates a large number of AGC protein kinase family members to control cellular responses and in physiological processes such as cell growth, proliferation and survival. GSK3β is an important target molecule downstream of AKT. It is generally believed that GSK3β is a tumor suppressor, and the activity of tumor cells is related to the inhibition of GSK3β, and is also a downstream molecule of PI3K/AKT pathway.

Recently, Xi et al. have shown that UFM1 can inhibit the sensitivity of endothelial cells to LPS through the NF-κB signaling pathway [12]. UFM1 is currently known to be closely related to several human diseases, including tumors. Although UFM1 has been reported to be involved in the progression of breast cancer, its role in gastric cancer is still unclear [13]. Our previous study has shown that the expression level of UFM1 is significantly down regulated in gastric cancer tissues than in adjacent tissues. Further analysis revealed a low expression level of UFM1 in gastric cancer, which was positively correlated with the TNM staging and poor prognosis of patients, suggesting that UFM1 may be a potential tumor suppressor gene in gastric cancer. We further studied the effects of UFM1 on gastric cancer function and in-depth mechanisms through a series of in vitro and in vivo experiments, aiming to clarify the relationship between UFM1 and the development of gastric cancer, and provide a new theoretical basis for early intervention and potential targeted treatment of gastric cancer.

## Materials and methods

### Human tissues

Gastric cancer specimens and the respective adjacent non-tumor tissues (within a minimum distance of 5 cm from the excised tumor) of 236 patients were obtained from the Department of Gastric Surgery, Fujian Medical University Union Hospital with detailed clinic pathologic parameters and detailed follow-up information from January 2013 to January 2018. All patients with gastric cancer had not received adjuvant chemotherapy before surgery and underwent gastrectomy with D2 lymph node (LN) dissection. Clinicopathological characteristics included age, gender, tumor size, tumor location, differentiation, histological type, depth of invasion, LN metastasis, distant metastasis, and TNM stage. T stage, N stage, and TNM stage were determined by using the 8th edition of the AJCC staging system [14] . Collected tissue samples were immediately frozen in liquid nitrogen and stored at − 80 °C until further analysis. Among them, samples from 116 patients (collected by gastrectomy from 2017 to 2018) were subjected to Western blotting and RT-qPCR analysis. Samples from the other 120 patients (collected by gastrectomy in 2013 to 2014) were collected for immunohistochemical staining. This study was approved by the ethics committee of Fujian Medical University Union Hospital, and written consent was obtained from all involved patients.

### Follow-up

All patients were followed up every 3 months for the first 2 years and every 6 months for the next 3–5 years. The last follow-up time point was January 2018. Routine examinations of follow-up, including a physical examination, laboratory tests (CA19–9, CEA and CA72–4), chest X-ray, abdominal CT, B ultrasound, and gastroscopy were performed each year. Overall survival was defined as the time from surgery to the date of death or to the last follow-up date, or to expiration of the follow-up (e.g., lost to follow-up, death from other diseases, etc.) The median follow-up interval was 37 months (range 0–62 months).

### Immunohistochemistry (IHC) and immunofluorescence

Immunohistochemistry (IHC) and immunofluorescence assays have been described previously [15]. Detailed methods are available in the Additional file 1.

### Cell culture and transfection

Human gastric cancer cell lines (N87, AGS, MGC-803 and HGC-27 cells) were purchased from the Institute of Biochemistry and Cell Biology, Chinese Academy of Sciences (Shanghai, China). Cells were cultured at 37 °C in a humidified atmosphere of 5% CO_2_ in 1640 (Gibco, Grand Island, NY) or DMEM/F12 1:1 medium containing 10% fetal bovine serum (FBS) (Gibco, Grand Island, NY).

Lentiviral constructs of UFM1 (NM_016617.4), shRNA (shUFM1), UFM1 overexpression (UFM1), and their corresponding empty vectors Lenti-scramble and Lenti-empty (shNCUFM1 and NCUFM1) were purchased from Shanghai Genechem Co. Ltd., China. The siRNA of PDK1 was purchased from Cell Signaling Technology (#6314). Cells were transfected with plasmid DNA using Lipofectamine 3000 and siRNA using Lipofectamine RNAiMAX transfection reagent (both from Thermo Fisher Scientific) following the manufacturer’s protocol. To establish a stable cell line, cells were selected after 48 h in a medium containing puromycin (1-2 mg/ml, Sigma) for at least 1 week after transfection, and cells were used for mRNA, protein analysis, and other assays. The sequences of shUFM1 were designed and chemically synthesized as follows: Forward,5′-CCGG-CCTGCTGCAACAAGTGCAATTCTCGAG AATTGCACTTGTTGCAGCAGG-TTTTTG-3′; Reverse,5′-AATTCAAAAA- ccTGCTGCAACAAGTGCAATTCTCGAGAATTGCACTTGTTGCAGCAGG-3′.

### Cell invasion and migration assay

A total of 2 × 10^4^ cells (100 μl cell suspension) were placed in a trans-well chamber, and 500 μl of culture medium containing 10% FBS was added to the lower chamber. After routine culture for 16–24 h, matrix and cells in the upper chamber were removed with a cotton swab. Cells were counted under a microscope after crystal violet staining.

### Quantitative real-time PCR

Total RNA was extracted from cell lines and tissue samples using TRIzol® reagent (Invitrogen, Carlsbad, CA, USA). First-strand cDNA was synthesized with PrimeScript RT Master Mix (Takara Biotechnology, Ltd., Dalian, China), according to the manufacturer’s protocol. After RT of total RNA, qPCR was conducted to examine the expression levels of UFM1 using SYBR Green PCR Master mix (Takara Biotechnology, Ltd.) on a Bio-Rad Real-Time PCR instrument (Bio-Rad Laboratories, Inc., Hercules, CA, USA). GAPDH was used as an internal reference gene to normalize the mRNA levels between different samples for an exact comparison of transcription levels. The sequences of the primers are provided in Additional file 2: Table S1. Data were analyzed using the ΔΔCt method [16] with GAPDH as the constitutive marker.

### Western blot assay

Samples and cells were collected for Western blotting as previously described [17]. Western blot analysis was performed using the following antibodies: PDK1 (#13037), Snail (#3879), p-AKT (#4060), AKT (#4685), GSK3β (#12456) and pGSK3β (#5558) were purchased from Cell Signaling Technology (Danvers, MA); UFM1 (ab109305), β-catenin (ab6302), GAPDH (ab8245), N-cadherin (ab18203), E-cadherin (ab1416), Vimentin (#92547), PI3KP110 and PI3KP85 (ab127617) were from Abcam (Cambridge, UK). Detailed methods are available in the Additional file 1.

### Human Phospho-kinase Array

The relative levels of protein phosphorylation were tested using the Human Phospho-Kinase Array Kit (ARY003B, R&D Systems, Inc. USA & Canada) according to the manufacturer’s protocol. An equal amount of protein (600 mg) was extracted from stable cells (shNCUFM1 AGS and shUFM1 AGS) and used to compare the kinase activity of UFM1 with and without UFM1 knockdown.

### Immunoprecipitation and in vitro ubiquitination assay

Cells were washed with ice-cold PBS and lysed using Ip pyrolysis solution containing a proteinase inhibitor cocktail tablet (Roche; 1 mM PMSF; 2 mM nethylmaleimide), using a pestle for homogenization. Lysates were incubated on ice for 30 min before cellular debris and nuclei were removed by centrifugation at 12,000 g for 15 min. Cell lysates were incubated with the corresponding primary antibody (PDK1 or UFM1) overnight at 4 °C. Protein A–Sepharose (Amersham Biosciences, Piscataway, NJ) beads in a 50:50 mixture of 50 mmol/L Tris buffer, pH 7.0 were added and further incubated for another 4 h at 4 °C. After elution, the proteins were separated by SDS-PAGE followed by Western blot analysis using the corresponding antibody. For the in vivo ubiquitination assay, the cell lysate (an equal amount of protein extracted from stable cells for the ubiquitination analysis) extracted from UFM1 knockdown or overexpression AGS cells was immunoprecipitated with anti-PDK1 antibody, and the ubiquitination level of PDK1 was tested with an anti-Ub antibody.

### Tumor xenograft transplantation assay

Male BALB/c nude mice (age, 4–5 weeks; weight, 14–16 g; Institute of Zoology, Chinese Academy of Sciences) were randomly divided into two groups: Negative control (NC) and UFM1 overexpression groups (*n* = 5/group). All animal procedures were performed according to the Animal Protection Committee of Fujian Medical University (Fuzhou, China), and the animal study was approved by the Ethics Committee of Fujian Medical University/Laboratory Animal Center (Fuzhou, China). The permission number is FJMUIACUC, 2019–0014. Part of the mice were primed with an injection of 1 × 10^7^ stable HGC-27 cells in 100 μl of phosphate-buffered saline (PBS) into the right axillary fossa of nude mice. The tumor volume was measured every 7 days and evaluated as follows: (length×width^^2^)/2 cm^2^. The mice were sacrificed (cervical dislocation) on day 35 after injection, and the tumor weight was measured. The xenograft tumors were fixed and embedded in paraffin, followed by HE (Hematoxylin-eosin) staining.

### Metastasis experiment in nude mice

The other 10 BALB/c mice were randomly divided into two groups: Negative control (NC) and UFM1 overexpression groups (*n* = 5/group), mice were anesthetized by intraperitoneal injection of 1% so dium pentobarbital (40 mg/kg). In the middle of the dorsal aspect, the midline of the left iliac crest and the posterior tibial line, an incision of about 1.5 cm was taken into the abdomen to reveal the spleen. The needle was inserted along the longitudinal axis of the spleen, and the HGC-27 cell suspension with UFM1 overexpression or victor (1 × 10^6^ cells/mL) was injected. Put the spleen back in place and suture the abdominal wall in full thickness. Mice were inspected every 5 days for signs of disease, such as abdominal distension, tumor detectable by palpation. Six weeks after injection, mice were sacrificed (cervical dislocation), Metastasis nodes in liver were determined by counting the number of visible nodules in dissected livers.

### Statistical analysis

All the data were processed using the SPSS23.0 statistical software package and Prism 7.0 software (GraphPad). Continuous values are expressed as the mean ± standard deviation and analyzed using the Student’s t-test. Categorical variables were analyzed using the χ^2^ or Fisher’s exact tests. Univariate survival analysis was performed using the Kaplan-Meier method, and the curves were compared using the log-rank test. Multivariate analysis was performed using the Cox proportional hazards model to further evaluate all the significant prognostic factors that were found in the univariate analysis. The difference was considered statistically significant at *P* < 0.05.

## Results

### UFM1 is downregulated in gastric cancer

**Fig. 1:**
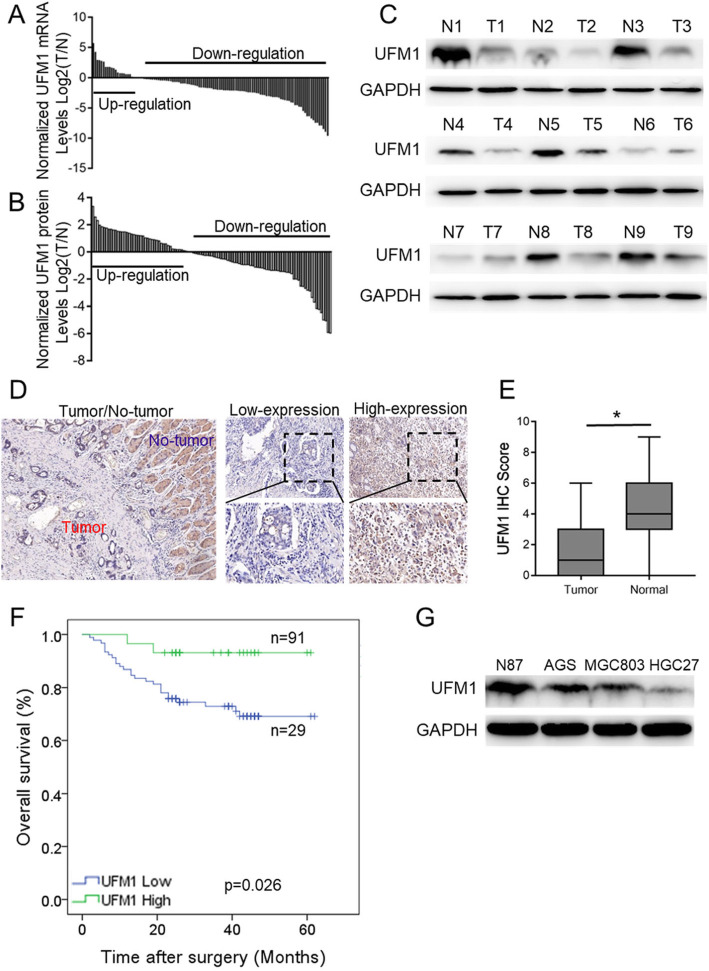
The expression and prognostic value of UFM1 in gastric cancer. **a** The mRNA levels of UFM1 in gastric tumors and respective adjacent tissues were measured by real-time qPCR. The ratios of UFM1 in gastric tumor compared to respective tissues (T/N) from 116 patients are presented. **b** The protein levels of UFM1 in gastric tumor tissues and respective adjacent tissues from 140 patients were measured using western blotting. The representative results are shown. **c** The T/N ratios of the total results described in (**b**). **d** The expression of UFM1 protein in gastric tumor tissues and respective adjacent tissues were analyzed using IHC (Representative results are shown, magnification, × 100 and × 400). **e** UFM1 expression scores are shown as box plots, with the horizontal lines representing the median; the bottom and top of the boxes representing the 25th and 75th percentiles, respectively; and the vertical bars representing the range of data. The expression of UFM1 in gastric tumor tissues and respective adjacent tissues was compared using the t-test. *n* = 120 (*, *P* < 0.005). **f** Kaplan Meier survival curve of gastric cancer patients with “High UFM1” or “Low UFM1” (*P* < 0.05, log-rank test). **g** Endogenous expression of UFM1 in various human gastric cancer cell lines by western blot

By qPCR analysis, we found that the transcription level of UFM1 was downregulated in gastric cancer tissues (*n* = 93, 80.2%) compared with the corresponding adjacent tissues (Fig. 1a). Using Western blot analysis, we confirmed that protein expression levels of UFM1 were significantly lower (*n* = 44, 41.9%) in tumor tissues compared with the corresponding adjacent nontumor tissues (*n* = 61, 58.1%, Fig. 1b, c) among 105 gastric cancer patients. In addition, we analyzed (Data mining from the published Oncomine database) the mRNA levels of UFM1 between normal and cancerous gastric tissue. The results showed that the transcription level of UFM1 in gastric cancer tissues was downregulated based on the data uploaded by Wang, consistent with the results of our present study (Additional file 3: Figure S1A).

In addition, we used immunohistochemistry to detect the expression of UFM1 in 120 paraffin-embedded tissues of gastric cancer (Fig. 1d, Additional file 3: Figure S1B) and then analyzed the effect of its expression on the prognosis of gastric cancer patients. The results showed that the expression of UFM1 was significantly higher in adjacent tissues than in the corresponding gastric cancer tissues (Fig. 1e). Among the 120 patients with primary gastric cancer, the UFM1 score was low in 91 cases (75.8%) and high in only 26 cases (24.2%), and the downregulation of UFM1 expression was closely related to the more advanced TNM stage (Table 1). Survival analysis showed that the 5-year survival rate of low UFM1 expression was 42.1%, which was significantly lower than the high UFM1 expression (*P* < 0.05, Fig. 1f). Additionally, we analyzed the effect of the UFM1 expression on the prognosis of gastric cancer patients according to the TCGA database [18]. The results also showed that patients with gastric cancer with high expression of UFM1 had a better prognosis (Additional file 3: Figure S1C). Concurrently, we also detected the expression level of UFM1 in several gastric cancer cell lines and found that the expression level of UFM1 was negatively correlated with the differentiation degree of gastric cancer cell lines (N87 is a highly differentiated gastric cancer cell, AGS and MGC-803 are moderately differentiated, HGC-27 is poorly differentiated) (Fig. 1g), suggesting that UFM1 may be closely related to the progression of gastric cancer.

**Table 1:** Relationships between UFM1 protein expressions in gastric cancer tissues and various clinicopathological variables

Variables	Total	UFM1 expression		P
		Low 91	High 29	
Gender				0.284
Male	95	70	25	
Female	25	21	4	
Age at surgery (years)				0.291
>60	80	63	17	
≤ 60	40	28	12	
Size of primary tumor (cm)				0.542
>5	48	35	13	
≤ 5	72	56	16	
Location of primary tumor				0.751
Lower 1/3	46	33	13	
Middle 1/3	19	16	3	
Upper 1/3	42	31	10	
More than 1/3	14	11	3	
Degree of differentiation				0.779
Well/moderate	47	35	12	
Poor and not	83	56	17	
Histological type				0.114
Papillary	54	43	11	
Tubular	47	23	14	
Mucinous	9	8	1	
Signet-ring cell	20	17	3	
Depth of invasion				0.077
T1+ T2	17	10	7	
T3+ T4	103	81	22	
Lymph node metastasis				0.283
N0	14	9	5	
N1–3	106	82	24	
TNM stage				0.027
I + II	38	24	14	
III + IV	83	67	15	
Distant metastasis				0.824
Negative	115	87	28	
Positive	5	4	1	

### UFM1 suppresses the invasion and metastasis of gastric Cancer cells

**Fig. 2:**
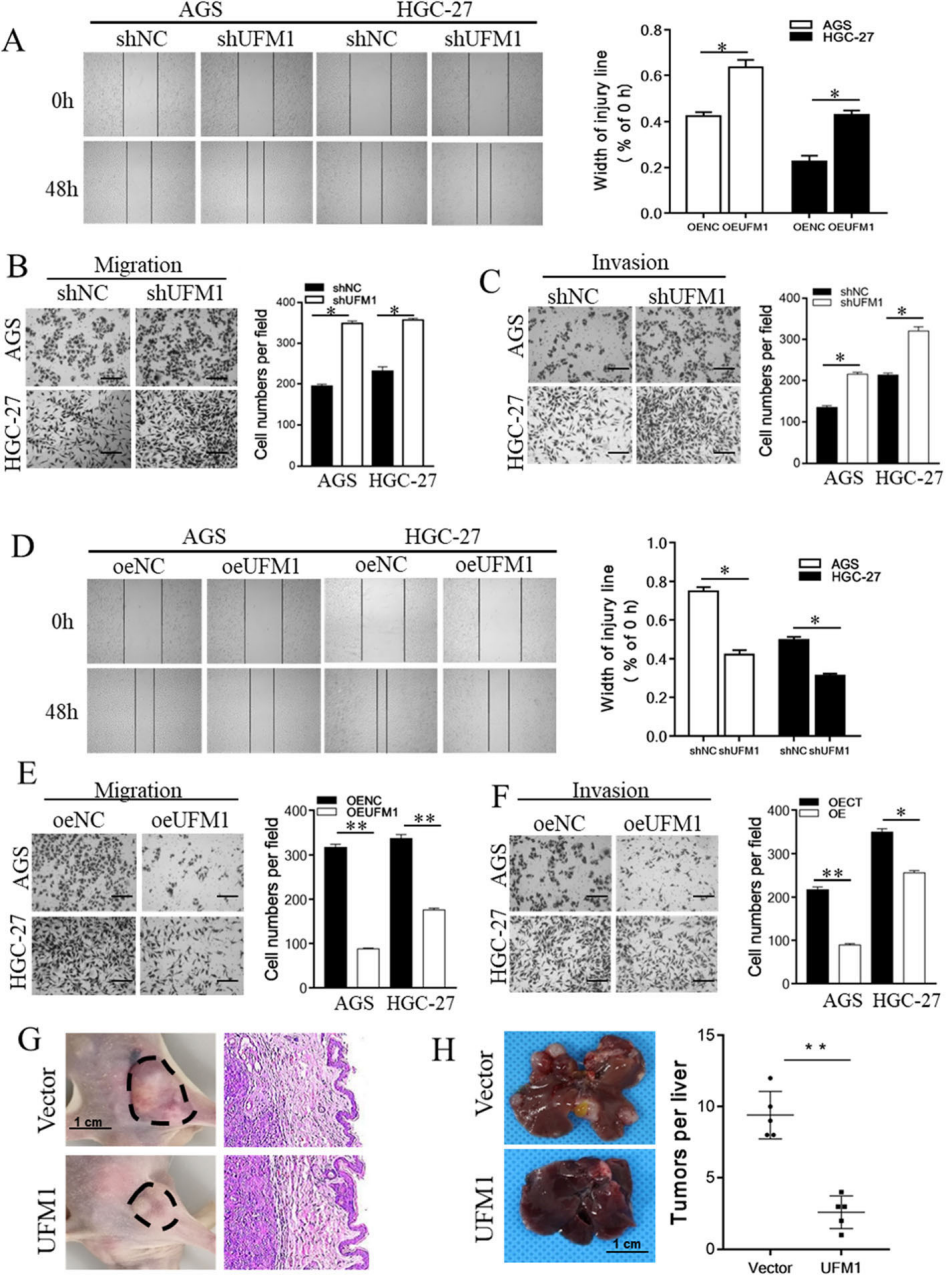
The expression of UFM1 was negatively correlated with the aggressive behaviors of gastric cancer cells in vitro and in vivo. **a** Wound-healing assays of stably down-regulated UFM1 expression in HGC-27 and AGS cells were performed. The representative images and the quantification were presented (*P < 0.05). **b** Migration assays of stably down-regulated UFM1 expression in HGC-27 and AGS cells were performed. The representative images and the quantification of the results are presented as mean ± SD; scale bar, 50 μm (* *P* < 0.05). **c** Invasion assays of stably down-regulated UFM1 expression in HGC-27 and AGS cells were performed. The representative images and the quantification of the results are presented as mean ± SD; scale bar, 50 μm (* *P* < 0.05). **d** Wound-healing assays of stably up-regulated UFM1 expression in HGC-27 and AGS cells were performed. The representative images and the quantification was presented (*P < 0.05). **e** Migration assays of stably up-regulated UFM1 expression in HGC-27 and AGS cells were performed. The representative images and the quantification of the results are presented as mean ± SD; scale bar, 50 μm (* *P* < 0.05). **f** Invasion assays of stably up-regulated UFM1 expression in HGC-27 and AGS cells were performed. The representative images and the quantification of the results are presented as mean ± SD; scale bar, 50 μm (* *P* < 0.05). **g** Gross photos of flank xenografts and microscopic photo leading edge of tumors. **h** Representative images of whole livers showing reduction in UFM1 liver tumour burden after infection with UFM1 compared to infection with control and showing a significant reduction in tumour burden after infection with UFM1 compared to control (*n* = 5 mice). **P < 0.01, two-sided unpaired t-test. Data are mean ± SD

To investigate the potential role of UFM1 in the invasion and metastasis of gastric cancer, we constructed HGC-27 and AGS gastric cancer cell lines with stable overexpressing or downregulation of UFM1 (Additional file 4: Figure S2A). We found that knockdown of UFM1 expression promoted the migration and invasion ability of these two cells in vitro (Fig. 2a, b, c); in contrast, upregulated UFM1 expression levels resulted in cell wound healing inhibition (Fig. 2d), as well as a significant reduction of the migration and invasion ability (Fig. 2e, f).

We also constructed a subcutaneous tumor model in nude mice to evaluate the effect of UFM1 on the tumorigenic ability of gastric cancer cells in vivo. After overexpression of UFM1, tumor growth was inhibited, and the growth of tumor volume was significantly decelerated compared with the control group (Additional file 4: Figure S2B). Moreover, the tumors were completely excised together with the skin, HE staining revealed that the tumor had significantly invaded the surrounding tissues in the control group, and the boundary with the surrounding tissues was unclear; However, when UFM1 was overexpressed, the boundary between the tumor and the surrounding tissue was clearly visible (Fig. 2g). In addition, we also performed a nude mouse liver metastasis model experiment. Stable HGC-27 cells which overexpression UFM1 was injected into the spleen of 8-week-old nude mice. Tumor burden was assessed at 16 weeks of age. Macroscopic examination, the representative images of whole livers showing reduction in UFM1 liver tumor burden after infection with UFM1 compared to infection with control (Fig. 2h). These results indicate that UFM1 not only inhibits the tumorigenic ability of gastric cancer cells but also significantly reduces the invasion ability of gastric cancer cells.

### UFM1 inhibits the invasion and metastasis of gastric Cancer through EMT

**Fig. 3:**
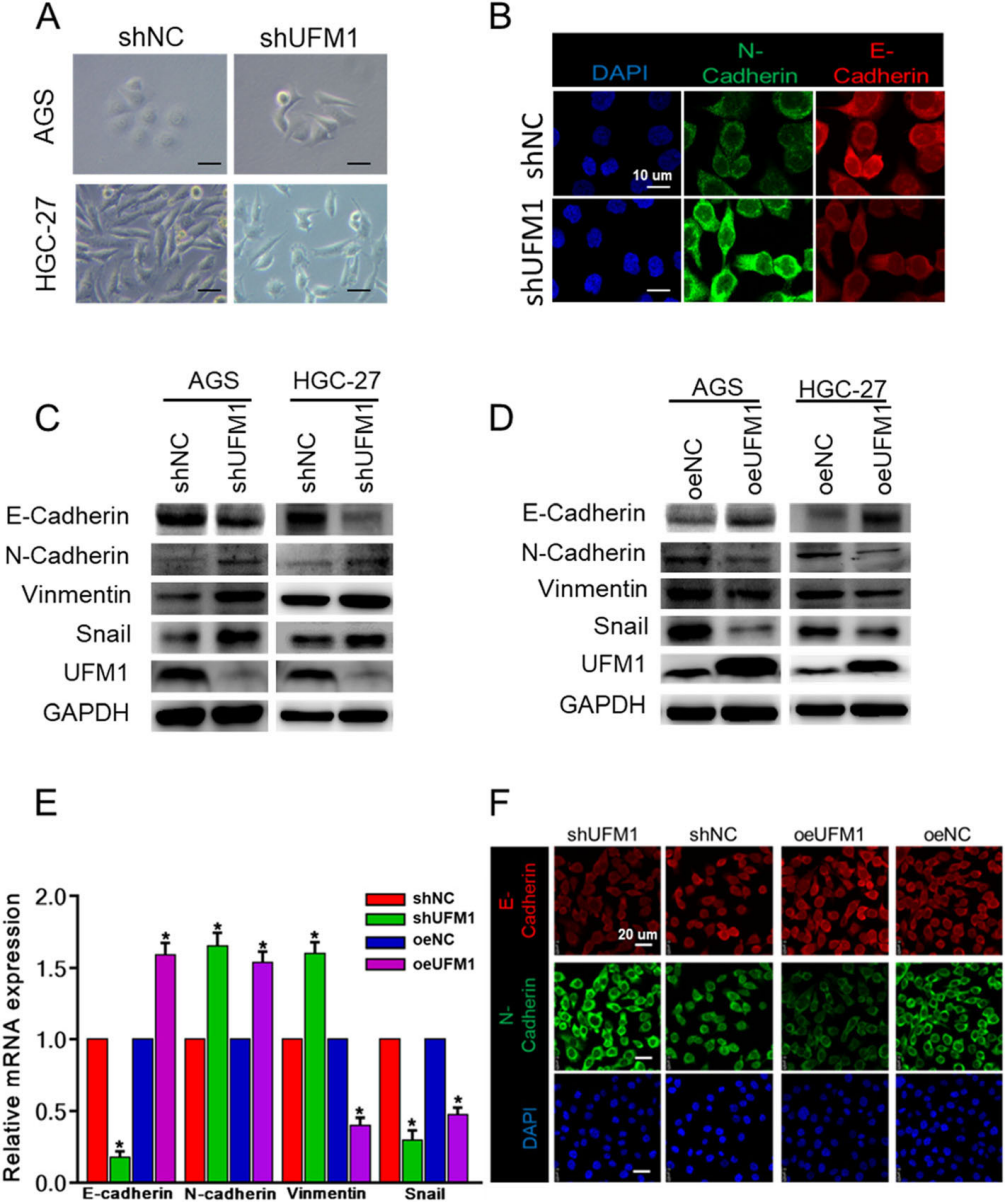
UFM1 suppresses the metastatic potential and epithelial-to-mesenchymal transition of gastric cancer. **a** Representative images of cell morphology in stable HGC-27 and AGS cell expressing control shRNA (shNC) or UFM1 shRNA; scale bar, 50 μm. **b** Representative images of cell morphology in stably down-regulated UFM1 expression in AGS cell by immunofluorescence staining. **c** The lysates of stably down-regulated UFM1 expression in HGC-27 and AGS cells were respectively applied to western blot to detect the expression levels of E-cadherin, N-cadherin, vimentin, Snail and UFM1. **d** The lysates of stably up-regulated UFM1 expression in HGC-27 and AGS cells were respectively applied to western blot to detect the expression levels of E-cadherin, N-cadherin, vimentin, Snail and UFM1. **e** Stable HGC-27 cells were applied to real-time PCR. Data shown were the log values of the fold-changes in mRNA levels as compared to control. **f** Stable AGS cells were applied to immunofluorescence staining. Images shown were representatives in each group

Epithelial mesenchymal transition (EMT) makes cancer cells invasive and metastatic, and it plays a key role in tumor progression. We observed that knockdown of UFM1 resulted in a decrease in tight junctions between cells and a morphological change in epithelial mesenchymal transition in gastric cancer cells (Fig. 3a, b). We further examined the potential effect of UFM1 on EMT-related marker molecules. Western blot showed that E-cadherin expression was decreased and of N-cadherin, Vimentin, and Snail were increased in gastric cancer cells, which downregulated UFM1 expression (Fig. 3c). In contrast, overexpression of UFM1 had an opposite effect on the related proteins (Fig. 3d). These results were also confirmed at the transcriptional level (Fig. 3e). Furthermore, we used immunofluorescence to detect the expression levels of E-cadherin and N-cadherin in AGS cells with stable overexpression and knockdown of UFM1, the results also consistent with the findings obtained by Western blot (Fig. 3f).

### UFM1 suppresses epithelial-to-mesenchymal transition of gastric cancer by inactivating the PI3K/AKT pathway

**Fig. 4:**
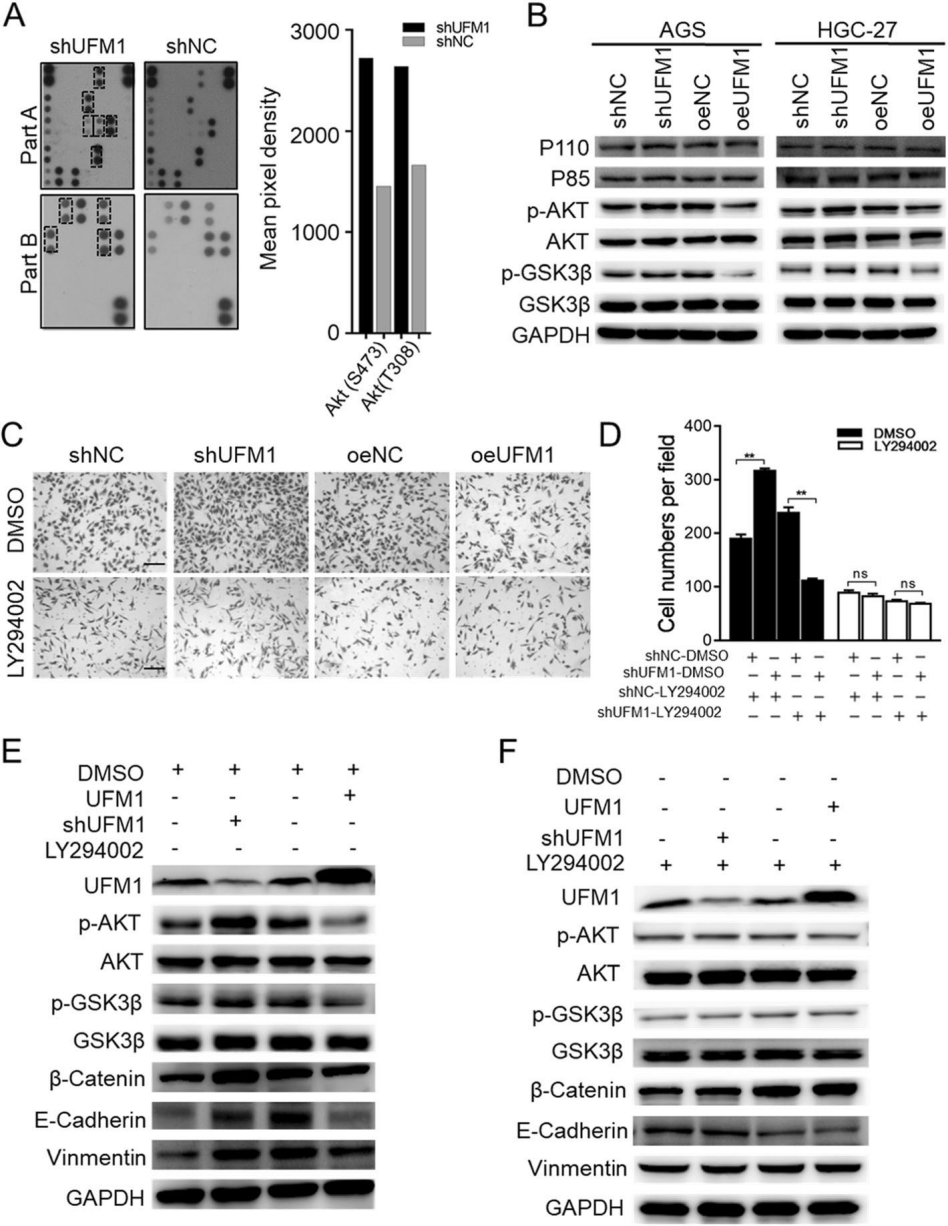
UFM1 suppresses epithelial-to-mesenchymal transition of gastric cancer through inactivating AKT/GSK-3β pathway. **a** The lysates of stable AGS cells were applied to Phospho-Kinase Antibody Array, and pixel densities of indicated proteins were shown in the right panel. **b** The lysates of stable AGS cells were applied to western blot to detect the expression levels of P110, P85, p-AKT, AKT, p-GSK3βand GSK3β. **c** The effect of UFM1 stable expression on AGS and HGC-27 cell migration was rescued by LY294002. **d** Quantitative results of (**c**) is show. The data are presented as the mean ± SD; scale bar, 50 μm (**, *P* < 0.01; ns, no significance). **e** Stable AGS cells were treated with DMSO or LY294002. Then cell lysates were applied in western blot analysis

To explore the mechanism of UFM1 on the invasion and metastasis of gastric cancer, we screened 10 differentially regulated signaling molecules in AGS (downregulated UFM1 expression levels) using the human phosphokinase microarray. We found that the levels of p-AKT (S473) and p-AKT (T308) were significantly increased (Fig. 4a, Additional file 5: Figure S3A). Next, we detect the expression levels of PI3K/AKT/GSK3β-related proteins in AGS and HGC-27 cells stably transfected with UFM1. Over-expression of UFM1 inhibited the phosphorylation of AKT and GSK3β. Phosphorylation of AKT and GSK 3β increased following the downregulation of UFM1 expression, but there was no significant difference in protein levels between P110 and P85 (Fig. 4b). This result indicates that UFM1 can affect the PI3K/AKT signaling pathway by affecting the phosphorylation of AKT. Subsequently, we used the PI3K inhibitor LY294002 to assess whether it could inhibit the invasion phenotype of gastric cancer (Additional file 5: Figure S3B). Further studies showed that LY294002 significantly inhibited the phosphorylation of AKT, but the expression level of UFM1 did not significantly change. However, the phosphorylation level of AKT was significantly increased after knocking down UFM1 (Additional file 5: Figure S3C). Therefore, we propose that UFM1 functions upstream of AKT. In addition, we also found that LY294002 blocked the effect of cell (stable transfection of UFM1) migration (Fig. 4c, d) and the phosphorylated AKT/GSK3β and EMT-related protein levels (Fig. 4e). Based on the above findings, we believe that UFM1 affects the phosphorylation level of AKT/GSK3β and EMT in gastric cancer cells, but it does not affect PI3K.

### UFM1 suppresses the metastatic potential and EMT of gastric cancer in a PDK1-dependent manner

**Fig. 5:**
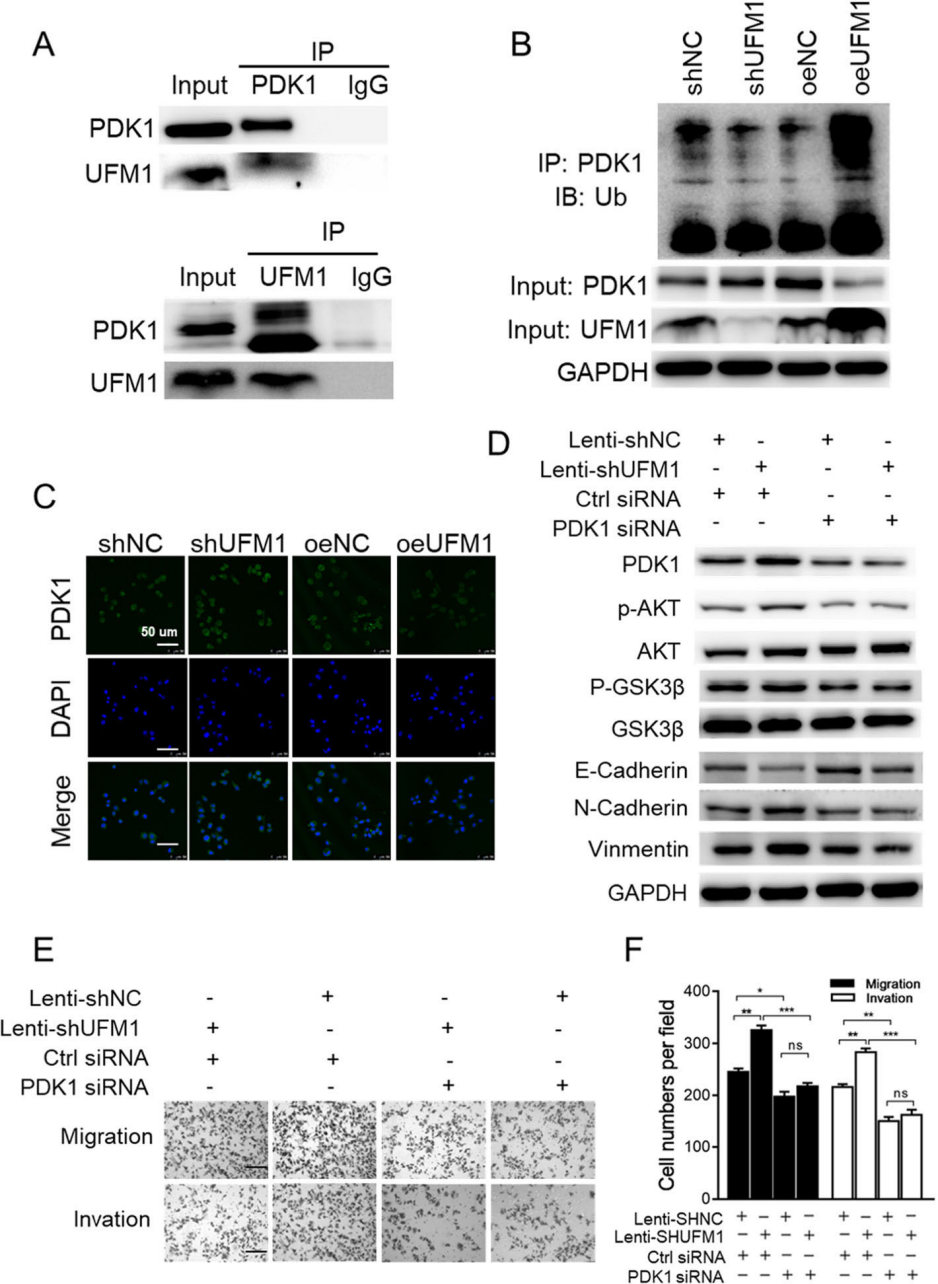
UFM1 suppresses the metastatic potential and epithelial-to-mesenchymal transition of gastric cancer in PDK1-dependent manner. **a** UFM1 associates with PDK1 in gastric cancer. Immunoprecipitation using PDK1 antibody was performed in AGS cell lysates (up panel). Stable AGS cells (down panel) were collected, lysed, and cell lysates were applied to immunoprecipitation with UFM1 antibody. **b** UFM1 promoted PDK1 ubiquitination. 293 T cells were cotransfected with constructs as indicated. PDK1 was immunoprecipitated with an anti-PDK1 antibody, and the ubiquitinated PDK1 was visualized by Western blot analysis using an anti-Ub antibody. **c** Immunofluorescence images showing the changes in PDK1 in stable AGS cells. **d** Stable AGS cells were treated with Control siRNA or PDK1 siRNA then cell lysates were applied in western blot analysis. **e** The stimulatory effect of UFM1 downregulation on AGS cell migration and invasion was rescued by PDK1 siRNA transfection; scale bar, 50 μm. **f** Quantitative results of (**e**) is show. The data are presented as the mean ± SD (*P < 0.05; **P < 0.01; ns, no significance)

To further explore the possible mechanism by which UFM1 regulates the metastatic potential and EMT in gastric cancer, we used co-immunoprecipitation to identify possible binding proteins for UFM1. The results showed that UFM1 binds to PDK1 but not to E-cadherin, N-cadherin, PI3K, AKT or Snail (Fig. 5a, Additional file 6: Figure S4A). To further investigate the effect of UFM1 on PDK1 expression, we also searched the TCGA database and found no significant correlation between UFM1 and PDK1 at the transcriptional level [19] (Additional file 6: Figure S4B). Therefore, we hypothesized that UFM1 might regulate the protein level of PDK1 through post-translational modification. It has been reported in the literature that PDK1 contains a polyubiquitination modification [20]. Consequently, we examined the ubiquitination level of PDK1 after overexpression and knockdown of UFM1. When the expression level of UFM1 was decreased, the ubiquitination levels of PDK1 were decreased and the protein levels of PDK1 were elevated. Conversely, after overexpression of UFM1, the PDK1 ubiquitination level was elevated and protein levels reduced (Fig. 5b). Using the GeneMANIA website, we also found that the UFM1 modification system interacts with PDK1 (Additional file 6: Figure S4C), further validating our results [21]. Correspondingly, as the expression level of UFM1 gradually increased, the expression level of PDK1 decreased (Additional file 6: Figure S4D). In addition, our immunofluorescence results were consistent with the findings of the Western blot analysis (Fig. 5c).

We then investigated whether PDK1 could play a key role in UFM1-mediated gastric cancer metastasis inhibition. In AGS cells, we used PDK1 siRNA and observed a significant reduction of cell invasiveness (Additional file 6: Figure S4E). Furthermore, Western blot analysis showed that PDK1 siRNA could inhibit the downregulation of E-cadherin and the expression of N-cadherin and vimentin mediated by UFM1 in AGS (downregulated UFM1 expression) (Fig. 5d). In the trans-well assay, PDK1 siRNA blocked cell metastasis, which was inhibited by UFM1 (Fig. 5e, f). Subsequently, the related protein expression levels (proteins were extracted from subcutaneous tumor tissues) of the UFM1 overexpression group was consistent with the conclusions obtained from cell experiments (Additional file 6: Figure S4F). These results suggest that UFM1 inhibits cell migration, invasion and EMT in gastric cancer, and these phenomena are PDK1-dependent.

### Low expression of UFM1 and high expression of PDK1 indicate a poor prognosis in patients with gastric Cancer

**Fig. 6:**
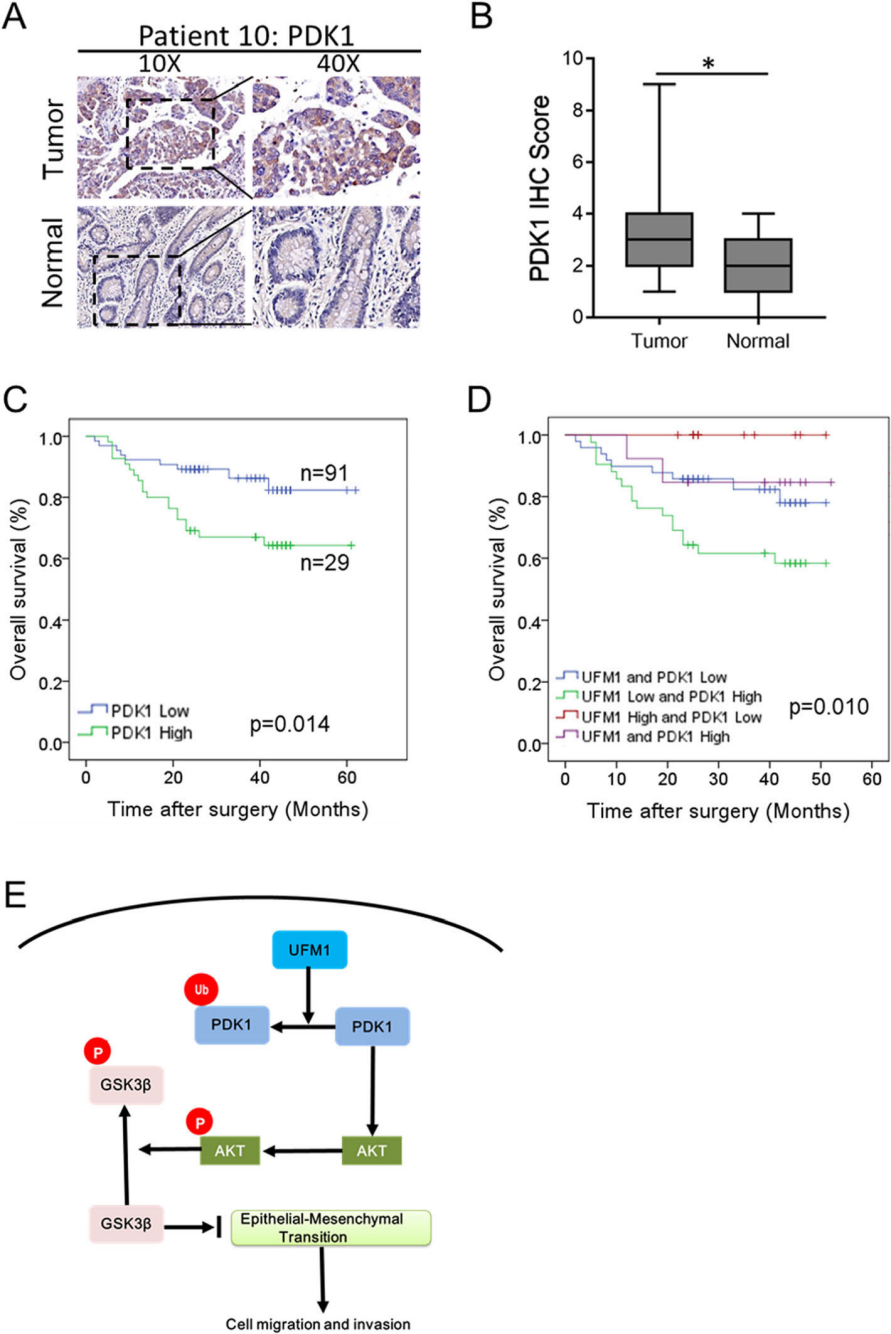
Effect of UFM1 and PDK1 on prognosis of patients with gastric cancer. **a** The expression of PDK1 protein in gastric tumor tissues and respective adjacent tissues were analyzed using IHC, magnification, × 100 and × 400. **b** PDK1 expression scores are shown as box plots. The expression of PDK1 in gastric tumor tissues and respective adjacent tissues was compared using the t-test. *n* = 120 (*, *P* < 0.005). **c** Kaplan-Meier survival curve of gastric cancer patients with “High PDK1” or “Low PDK1” (*P* < 0.05, log-rank test). **d** Kaplan-Meier analysis of the correlation between the combined expression of UFM1 and PDK1 with the overall survival of gastric cancer patients (*P* < 0.05, log-rank test). **e** Working model of the role of UFM1 in PDK1 signaling and gastric cancer cell invasion

In addition, we examined the protein level of PDK1 in paraffin-embedded tissue samples from 120 patients with gastric cancer (Additional file 7: Figure S5). The score for PDK1 was higher in gastric cancer tissues (*n* = 55, 45.8%) than the corresponding adjacent tissues (Fig. 6a, b). The survival analysis showed that patients with high expression of PDK1 had worse survival rates than those with low expression of PDK1 (64.3% vs. 82.3%, *p* = 0.014, Fig. 6c). Combined with the co-expression of UFM1 and PDK1, we found that patients with low expression of UFM1 and high expression of PDK1 had the worst prognosis (58.4%), while those with high expression of UFM1 and low expression of PDK1 had the best prognosis (Fig. 6d). We used a Cox proportional hazards regression model to analyze the effects of UFM1 and PDK1 expression and other clinicopathological data on the prognosis of patients with gastric cancer. Univariate analysis showed that depth of invasion, lymph node metastasis, TNM stage, distant metastasis, UFM1 expression, PDK1 expression, and combined with UFM1 and PDK1 expression were closely related to patient prognosis (Table 2). Multivariate Cox regression analysis showed that co-expression of UFM1 and PDK1 and TNM stage were both independent predictors for gastric cancer (Table 3).

**Table 2:** Univariate analysis of the correlation between clinicopathological parameters and survival of patients with gastric cancer

Clinicopathological parameters	Three-year cumulative survival rate	Log-Rank Test	*p*
Gender			
Male	72.7	0.096	0.756
Female	80.0		
Age (years)			
> 60	75.6	0.102	0.749
≤ 60	70.5		
Tumor size (cm)			
> 5	69.9	1.435	0.231
≤ 5	76.8		
Location of tumor			
Lower 1/3	73.3	3.943	0.268
Middle 1/3	82.6		
Upper 1/3	66.8		
More than 1/3	92.9		
Degree of differentiation			
Well/moderate	78.7	0.332	0.564
Poor and not	70.8		
Histological type			
Papillary	65.4	3.825	0.281
Tubular	83.6		
Mucinous	66.7		
Signet-ring cell	85.0		
Depth of invasion			
T1 + T2	100.0	5.357	0.021
T3 + T4	70.1		
Lymph node metastasis			
Negative	100.0	4.453	0.035
Positive	70.7		
TNM stage			
I + II	97.4	12.542	0.000
III + IV	63.2		
Distant metastasis			
Negative	75.9	6.798	0.009
Positive	40.0		
UFM1 expression			
Low	69.1	4.946	0.026
High	93.1		
PDK1 expression			
Low	82.3	5.990	0.014
High	64.3		
UFM1/PDK1 expression			
UFM1 high and PDK1 low	100.0	11.290	0.010
UFM1 and PDK1 high	84.6		
UFM1 and PDK1 low	78.0		
UFM1 low and PDK1 high	58.4

**Table 3:** Multivariate analysis of the correlation between clinicopathological parameters and survival time of patients with gastric cancer

Covariates	Coefficient	Standard error	HR	95% CI for HR	*p*
UFM1 expression (high vs. low)	−1.226	0.735	0.294	0.070–1.239	0.095
PDK1 expression (high vs. low)	−0.979	0.536	0.376	0.131–1.074	0.068
UFM1 and PDK1 expression (low/low vs. high and/or high)	1.178	0.453	3.247	1.336–7.891	0.009
Depth of invasion (T3,T4 vs. T1,T2)	1.071	1.045	2.920	0.376–22.635	0.305
Lymph node metastasis (positive vs. negative)	1.538	1.020	1.974	0.631–34.367	0.132
Distant metastasis (positive vs. negative)	0.861	0.544	2.365	0.815–6.8631	0.113
TNM stage (stage III and IV vs. I and II)	−1.630	0.608	7.195	0.060–0.645	0.007

## Discussion

Previous studies of UFM1 have focused on the pathophysiological effects of the endoplasmic reticulum stress response. However, whether UFM1 is expressed in epithelial cells and its biological function are still unclear. In the present study, we found that UFM1 was downregulated in gastric cancer tissue. Gastric cancer patients with low expression of UFM1 presented a poor prognosis. UFM1 had an inhibitory effect on the tumorigenicity, invasion and migration of gastric cancer cells. Further studies have shown that UFM1 can inhibit the phosphorylation of AKT and downstream GSK3β by binding to PDK1 and increasing its ubiquitination, thereby inhibiting EMT of gastric cancer cells and exerting a tumor suppressor function.

EMT is an important biological process for the invasion and metastasis of epithelial-derived malignant tumor cells. It plays a key role in tumor invasion and metastasis, and it is an important step in the malignant progression of tumors [22, 23]. Changes in the cytoskeleton and related phenotypic genes are often associated with the development of EMT in epithelial tumor cells, such as a decrease in the epithelial marker E-cadherin and the interstitial marker N-cadherin, an increase in Vimentin or other protein involved in tight junctions, loss of polarity and morphological changes of mesenchymal cells, which result in decreased adhesion of tumor cells and an enhanced exercise capacity [24]. EMT activation is dependent on the stimulation of a variety of extracellular signals and the driving of EMT-inducible transcription factors such as Snail, ZEB, and Twist, which transform epithelial cells into a mesenchymal phenotype with migration and invasion abilities [25]. Multiple signaling pathways have been found to be involved in EMT, including the TGF-β/Smad, Wnt, Notch, ERK/MAPK, PI3K/AKT/GSK3β and NF-κB signaling pathways [25–27]. In this study, we found that UFM1 activated GSK3β by inhibiting the AKT/GSK3β pathway to regulate EMT. Interestingly, when we upregulated and downregulated the expression level of UFM1, there was no significant difference in protein levels between P110 and P85. Therefore, we considered that UFM1 does not affect the phosphorylation level of AKT/GSK3β by affecting PI3K. By using immunoprecipitation, we found that UFM1 and PDK1 could bind to each other. When UFM1 was overexpressed, the expression level of PDK1 was decreased, and when the opposite UFM1 was weakened, the expression level of PDK1 was increased.

The PDK1 molecule contains 36 lysines, 27 of which are located in the N-terminal kinase domain and 9 of which are located in the C-terminal platelet-leukocyte C kinase substrate homolog domain (C-terminal pleckstrin homology domain) [28, 29]. PDK1 belongs to the AGC protein kinase family (cAMP and cGMP-dependent protein kinase C), which phosphorylates many downstream proteins such as AKT, p90RSK, p70S6K, SGK and PKC [28, 30–32]. Studies have shown that blocking the interaction between PDK1 and AKT can inhibit the growth and metastasis of melanoma [33]. Therefore, we used siRNA to knockdown PDK1 in AGS cells with stable downregulated expression of UFM1. In our study, we thought that using the same cell line for overexpression and knockdownof the target gene for cell function assay could avoid the interference of cell line changes on the experimental results, and it can also reflect the effect of genetic changes on the function of the cell line. We found that also in many literatures also overexpressed and knocked out using the same cell line [34, 35]. We tested the expression level of UFM1 protein in different gastric cancer cell lines and found that AGS was at a medium level. Therefore, we mainly chose AGS for overexpression and knockdownthen for cell function experiments. In addition, we verified these results in HGC-27 gastric cancer cells. Although the expression level of CDK5RAP3 in HGC-27 was low, but the experiment after knocking down and overexpression the target gene also reflects the effect of the gene on cell function. Furthermore, we found that the inhibitory effect of UFM1 on gastric cancer cell EMT was also prevented when PDK1 was knocked down. Studies by Paolo Armando et al. have shown that PDK1 deletion can affect the EMT process in endothelial cells [36]. Alfonso et al. have shown that PDK1-Akt signaling pathway activity is directly related to EMT, and inhibition of the PDK1-Akt signaling pathway can transform endothelial cells in the embryonic atrioventricular pathway into mesenchymal cells, which is similar to our findings [37]. In addition, we also demonstrated that UFM1 could inhibit PDK1 expression at the post-translational level. When UFM1 expression was upregulated, the level of ubiquitination of PDK1 was decreased, and the level of PDK1 protein was increased. Conversely, when UFM1 expression was decreased, the level of ubiquitination of PDK1 was increased, and the protein levels were decreased. Hak Ha et al. reported that estrogen receptor α can promote the transcriptional activation of estrogen receptor and growth of breast cancer by competitively binding to the UFM1-specific protease UfSP2 bound to the ASC1 zinc finger structure [7]. In another study, UFM1 was observed to interact with IKKβ protein and ubiquitinate-modify IKKβ, thus enhancing the transcriptional activity of NF-κB, which is involved in NF-κB signaling pathway regulation [38]. In this study, we found that (through the Gene MANIA public database) the modification system of UFM1 interacted with PDK1 (Additional file 6: Figure S4C). Furthermore, in the clinical specimens from patients, the expression of UFM1 and PDK1 had a significant effect on the prognosis of patients. Patients with low expression of UFM1 and high expression of PDK1 had the worst prognosis. Therefore, we believe that UFM1 interacts with PDK1 and undergoes ubiquitination to reduce its expression level, which in turn reduces the phosphorylation level of AKT/GSK3β, inhibiting EMT. Our findings provide a novel mechanism for the tumorigenic activity of PDK1 in epithelial-derived cancer cells.

## Conclusion

In summary, our study shows that UFM1 may downregulate the expression level of PDK1, which inhibit the AKT/GSK3β pathway and downregulating the EMT activity of gastric cancer cells, leading to suppress the invasion and metastasis of gastric cancer cells. These findings suggest that UFM1 may be a potential new marker for the treatment of gastric cancer. The content of this study is summarized in a simplified schematic diagram (Fig. 6e).

### Supplementary information

**Additional file 1.** Materials and Methods.

**Additional file 2: Table S1.** Primer sequence.

**Additional file 3: Figure S1.** (A) Immunohistochemical staining of UFM1 expression in gastric cancer tissue and the criteria for immunohistochemistry scores following the intensity of positive signals, magnification, × 100. (B) Oncomine data mining analysis of UFM1 levels in Wang datasets between normal tissues versus gastric cancer. (C) Kaplan Meier curves of OS in GC patients with high or low UFM1 expression in TCGA-STAD. OS curves were generated by setting median UFM1 expression as cutoff. Analysis was performed using the UALCAN browser.

**Additional file 4: Figure S2.** (A) AGS and HGC-27 cells with stably overexpressed or knocked-down UFM1 were created. The UFM1 expression changes were confirmed by western blotting. (B) Tumor volume of the xenografts was measured every 7 days.

**Additional file 5: Figure S3.** (A) The lysates of stable AGS cells were applied to Phospho-Kinase Antibody Array, and 10 pixel densities of indicated proteins were shown. (B) PI3K inhibitor LY294002 can inhibit the invasion phenotype of AGS and HGC-27 cell; scale bar, 50 μm. (C) LY294002 significantly inhibited the phosphorylation level of AKT, but the expression level of UFM1 did not change significantly. The phosphorylation level of AKT was significantly increased after knocking down UFM1.

**Additional file 6: Figure S4.** (A) The lysates of AGS cells were applied to immunoprecipitation using UFM1 antibody. The immunoprecipitates were examined to blot PI3K subunits p85 and p110, AKT, EMT-related proteins E-cadherin, N-cadherin and Snail. (B) The relationship of UFM1 and PDK1 in mRNA by Linkedomics browser. There was no obvious correlation between them (*P* = 0.314). (C) UFM1 modification system could interacts with PDK1 by the GeneMANIA browser. (D) AGS cells were transfected as indicated then applied to western blot. (E) PDK1 siRNA significant reduce AGS cell invasiveness. The data are presented as the mean ± SD; scale bar, 50 μm (*P < 0.05).

**Additional file 7: Figure S5.** (A) Immunohistochemical staining of PDK1 expression in gastric cancer tissue and the criteria for immunohistochemistry scores following the intensity of positive signals, magnification, × 100.

## Data Availability

All data generated during this study are included in this article.

## Abbreviations

AJCC: American joint committee on cancer
AKT: Protein kinase B (PKB)
EMT: Epithelial-mesenchymal transition
GAPDH: Glyceraldehyde-3-phosphate dehydrogenase
GSK3β: Glycogen synthase kinase 3 beta
LPS: Lipopolysaccharides
NF-κB: Nuclear factor kappa-light-chain-enhancer of activated B cells
PCR: Polymerase Chain Reaction
PDK1: Protein 3-phosphoinositide-dependent protein kinase-1
PI3K: Phosphatidylinositol 3-kinases
TCGA: The cancer genome atla
TNM: Tumor-node-metastasis
UFC1: Ubiquitin Fold Modifier Conjugating Enzyme 1
UFL1: UFM1-protein ligase 1
UFM1: Ubiquitin-fold modifier 1
