# Correction: UFM1 suppresses invasive activities of gastric cancer cells by attenuating the expression of PDK1 through PI3K/AKT signaling

**DOI:** 10.1186/s13046-023-02896-7

**Published:** 2023-11-21

**Authors:** Jian-Xian Lin, Xin-Sheng Xie, Xiong-Feng Weng, Sheng-Liang Qiu, Changhwan Yoon, Ning-Zi Lian, Jian-Wei Xie, Jia-Bin Wang, Jun Lu, Qi-Yue Chen, Long-Long Cao, Mi Lin, Ru-Hong Tu, Ying-Hong Yang, Chang-Ming Huang, Chao-Hui Zheng, Ping Li

**Affiliations:** 1 Department of Gastric Surgery, Fujian Medical University Union Hospital, Fuzhou, Fujian Province 350001 China; 2 Key Laboratory of Ministry of Education of Gastrointestinal Cancer, Fujian Medical University, Fuzhou, Fujian Province 350108 China; 3 Fujian Key Laboratory of Tumor Microbiology, Fujian Medical University, Fuzhou, Fujian Province 350108 China; 4 Department of Pathology, Fujian Medical University Union Hospital, Fuzhou, Fujian Province 350001 China; 5 Department of Surgery, Memorial Sloan Kettering Cancer Center, New York, NY USA

**Correction:** ***J Exp Clin Cancer Res*** **38, 410 (2019)**

10.1186/s13046-019-1416-4

Following the publication of the original article [1], minor errors were identified in the images of Fig. 5e, specifically:

- Invasion: Lenti-shUFM1+/Ctrl siRNA+.
- Invasion: Lenti-shNC+/Ctrl siRNA+.

The correct figure is given below:

**Fig. 5:**
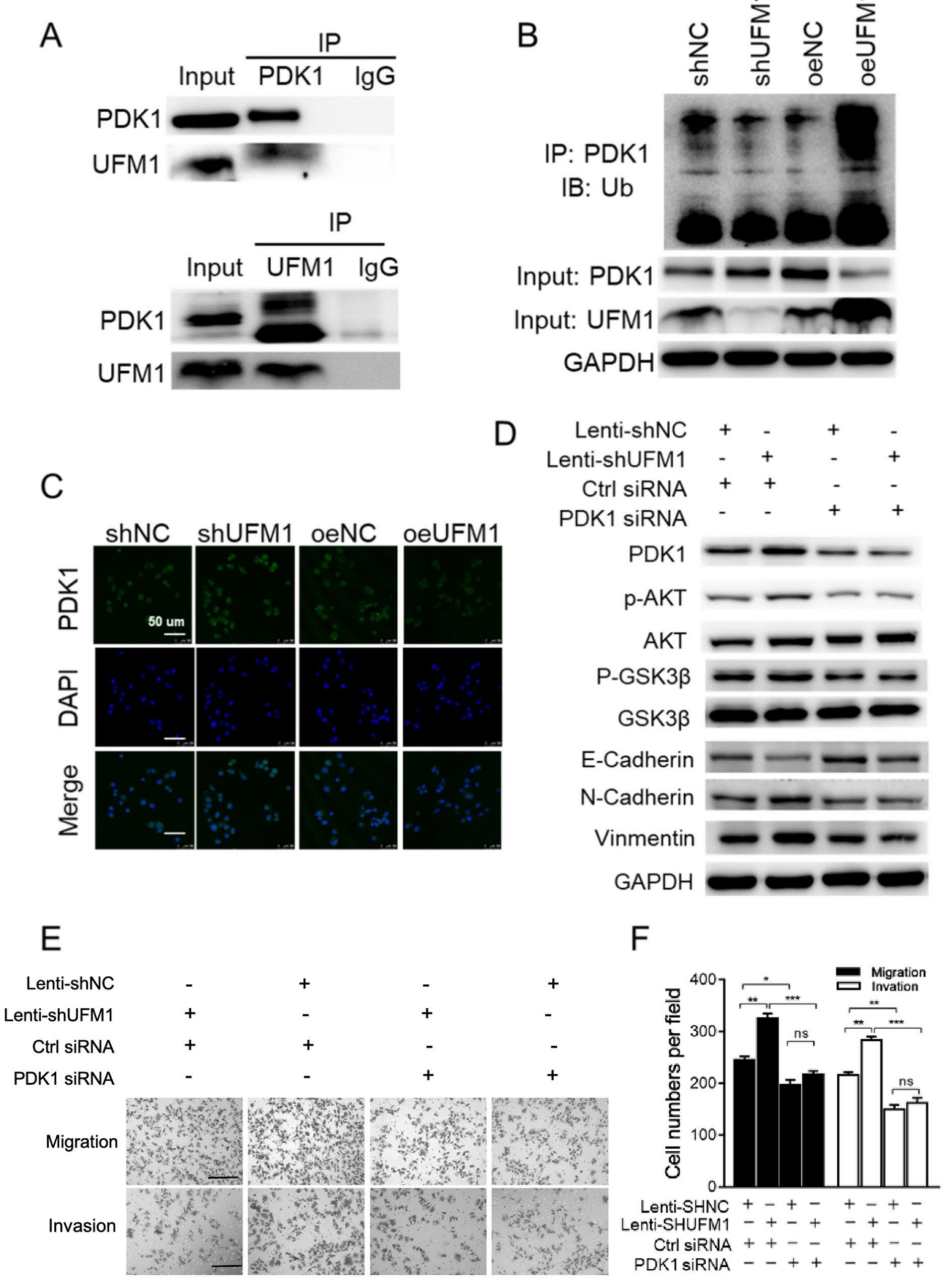
UFM1 suppresses the metastatic potential and epithelial-to-mesenchymal transition of gastric cancer in PDK1-dependent manner. **a** UFM1 associates with PDK1 in gastric cancer. Immunoprecipitation using PDK1 antibody was performed in AGS cell lysates (up panel). Stable AGS cells (down panel) were collected, lysed, and cell lysates were applied to immunoprecipitation with UFM1 antibody. **b** UFM1 promoted PDK1 ubiquitination. 293 T cells were cotransfected with constructs as indicated. PDK1 was immunoprecipitated with an anti-PDK1 antibody, and the ubiquitinated PDK1 was visualized by Western blot analysis using an anti-Ub antibody. **c** Immunofluorescence images showing the changes in PDK1 in stable AGS cells. **d** Stable AGS cells were treated with Control siRNA or PDK1 siRNA then cell lysates were applied in western blot analysis. **e** The stimulatory effect of UFM1 downregulation on AGS cell migration and invasion was rescued by PDK1 siRNA transfection; scale bar, 50 μm. **f** Quantitative results of (**e**) is show. The data are presented as the mean ± SD (*P < 0.05; **P < 0.01; ns, no significance)
