# Supplementary material for: UFM1 suppresses invasive activities of gastric cancer cells by attenuating the expression of PDK1 through PI3K/AKT signaling
Source: J Exp Clin Cancer Res. 2019 Sep 18;38:410. doi: 10.1186/s13046-019-1416-4 (PMC6751655; doi:10.1186/s13046-019-1416-4)
Supplement: Supplementary file 1 — Additional file 1. Materials and Methods. [file 13046_2019_1416_MOESM1_ESM.docx]

**Materials and Methods**

**Immunohistochemistry (IHC)**

Paraffin sections containing sufficient formalin fixed tumor tissue were sectioned continuously at a thickness of 4μm and mounted on silage-coated slides for immunohistochemical analysis. The slices were deparaffinized with xylene and rehydrated in 95%, 85% and 75% ethanol. Antigen retrieval was performed by subjecting the slides to high-pressure sterilization at 121°C for 2 min in 0.01mol/L sodium citrate buffer solutions (pH 6.0). Endogenous peroxidase activity was blocked by incubating the slides with 3% H_2_O_2_ at room temperature for 10 min. The sections were then washed with phosphate-buffered saline (PBS) solution and blocked in 10% goat serum (Zhongshan Biotechnology Co. Ltd.) for 30 minutes. Next, the sections were incubated with diluted rabbit anti-human UFM1, PDK1 (1:1000 dilution; Abcam; ab109305, 1:200 dilutions; Proteintech; 10026-1-AP) overnight in a humidified chamber at 4 °C. After three washes in PBS, the sections were incubated with the secondary antibody conjugated to horseradish peroxidase at room temperature for 30 minutes. The signal was developed with diaminobenzidine solution, followed by counterstaining in 20% hematoxylin. Finally, all slides were dehydrated and mounted on a cover glass. For the negative controls, nonspecific antibody diluent was substituted for the primary antibody.

IHC-stained tissue sections were reviewed by 2 experienced pathologists and independently scored based on stained cells and the intensity of staining. The standard for the evaluation of UFM1 and PDK1 staining intensity was as follows: no staining (score of 0), weak staining (score of 1), moderate staining (score of 2) and strong staining (score of 3). The positive proportion of stained tumor cells was scored as follows: ≤ 5% positive cells (score of 0), 6% to 25% positive cells (score of 1), 26% to 50% positive cells (score of 2), ≥ 51% positive cells (score of 3). If the total scores (percentage score × intensity score) was less than 3, the protein expression was considered low, however, if the score was 4 or higher, the protein expression was defined as high expression.

**Immunofluorescence**

The experimental steps of immunofluorescence were the same as those for immunohistochemistry until the first antibody was incubated. After an overnight incubation in a humidified box at 4 °C, PBS washing was performed three times, followed by the addition of fluorescein-labeled secondary antibodies and incubation at room temperature for 1 hour in the dark. The cells were washed three times with PBS in the dark. DAPI staining solution was added dropwise, and coverslips were mounted on a glass slide (Santa Cruz, USA) and incubated at room temperature for 1 minute in the dark. Images were obtained with a microscope (IX-70, Olympus, Japan).

**Western Blot Assay**

When the cells reached a confluency of 80-90%, they were flushed twice with precooled PBS and then extracted with RIPA pyrolysis solution (Thermo Fisher Scientific, Waltham, MA, USA) containing a 10% cocktail (phosphatase inhibitors, Roche, South San Francisco, CA, USA). In addition, protein was extracted from frozen gastric cancer and non-tumor tissues using a Whole Protein Extraction Kit (Fermentas Life Science, Glen Burnie, MD, USA) according to the manufacturer’s instructions. The protein concentration was determined using a BCA kit. Protein samples (40μg per lane) were separated on 10% polyacrylamide gels by SDS-PAGE and transferred to PVDF membranes. The PVDF membrane was then blocked with 5% skim milk at room temperature for 1 h. The membrane was incubated overnight at 4°C with primary antibody and washed 3 times with TBS-T (rinse buffer containing 0.05% Tween-20), 5 min each time, and incubated at room temperature with the HRP secondary antibody (Cell Signaling Technology) for 1 hour. GAPDH was used as an internal control. Finally, the membrane was washed with TBS-T for 30 minutes, and the protein bands were detected using an enhanced chemiluminescence method (Amersham Corporation, Arlington Heights, IL, USA).
