# Supplementary material for: UFM1 suppresses invasive activities of gastric cancer cells by attenuating the expression of PDK1 through PI3K/AKT signaling
Source: J Exp Clin Cancer Res. 2019 Sep 18;38:410. doi: 10.1186/s13046-019-1416-4 (PMC6751655; doi:10.1186/s13046-019-1416-4)
Supplement: Supplementary file 2 — Additional file 2: Table S1. Primer sequence. [file 13046_2019_1416_MOESM2_ESM.docx]

**Table S1 Primer sequence**

| Target gene | Accession number | Primer sequence (5'to3') |
| --- | --- | --- |
| UFM1 | NM_016617.4 | Forward: GTCCCCAGCACACTAGAGGA  Reverse: GGAAAAGAGCGGGAGAG AGT |
| GAPDH | NM_001289745.2 | Forward: TTGCCATCAATGACCCCTTCA  Reverse: CGCCCCACTTGATTTTGGA |
| E-cadherin | NM_004360.3 | Forward: TACACTGCCCAGGAGCCAGA  Reverse: TGGCACCAGTGTCCGGATTA |
| N-cadherin | NM_001792.3 | Forward: CAGTATCCGGTCCGATCTGC  Reverse: GTCCTGCTCACCACCACTAC |
| Vinmentin | NM_001292128.1 | Forward: CCACGAAGAGAAATCCAGG  Reverse: CAGAGAGGTCAGCAAACTTGG |
| Snail | NM_005985.3 | Forward: TCTGAGGCCAAGGATCTCCA  Reverse: TGGCTTCGGATGTGCATCTT |
