# Supplementary figures and images for: UFM1 suppresses invasive activities of gastric cancer cells by attenuating the expression of PDK1 through PI3K/AKT signaling
Source: J Exp Clin Cancer Res. 2019 Sep 18;38:410. doi: 10.1186/s13046-019-1416-4 (PMC6751655; doi:10.1186/s13046-019-1416-4)

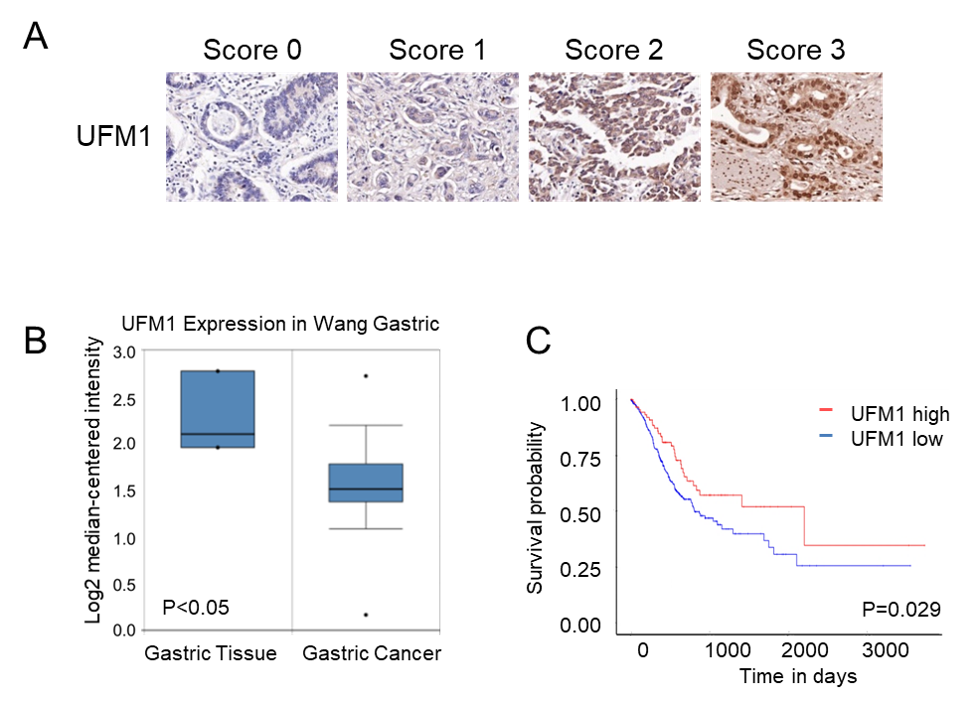

Supplement: Supplementary file 3 — Additional file 3: Figure S1. (A) Immunohistochemical staining of UFM1 expression in gastric cancer tissue and the criteria for immunohistochemistry scores following the intensity of positive signals, magnification, × 100. (B) Oncomine data mining analysis of UFM1 levels in Wang datasets between normal tissues versus gastric cancer. (C) Kaplan Meier curves of OS in GC patients with high or low UFM1 expression in TCGA-STAD. OS curves were generated by setting median UFM1 expression as cutoff. Analysis was performed using the UALCAN browser. [file 13046_2019_1416_MOESM3_ESM.tif]

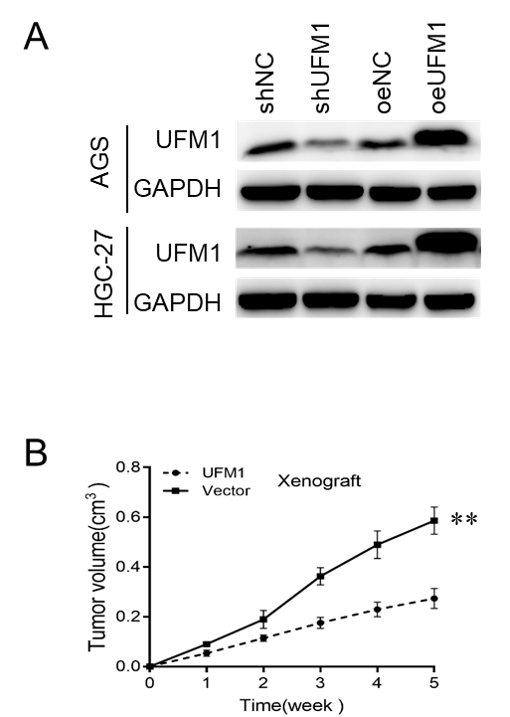

Supplement: Supplementary file 4 — Additional file 4: Figure S2. (A) AGS and HGC-27 cells with stably overexpressed or knocked-down UFM1 were created. The UFM1 expression changes were confirmed by western blotting. (B) Tumor volume of the xenografts was measured every 7 days. [file 13046_2019_1416_MOESM4_ESM.tif]

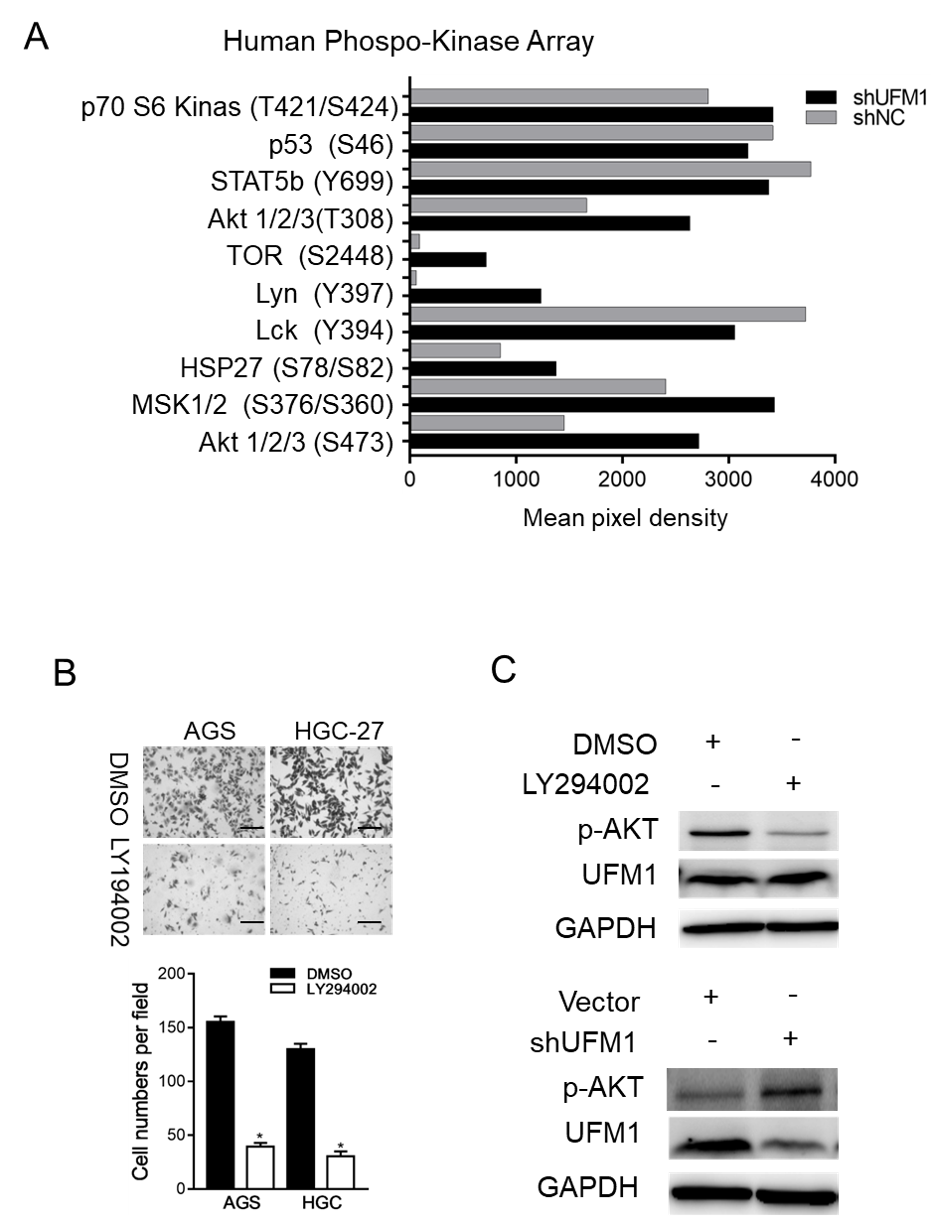

Supplement: Supplementary file 5 — Additional file 5: Figure S3. (A) The lysates of stable AGS cells were applied to Phospho-Kinase Antibody Array, and 10 pixel densities of indicated proteins were shown. (B) PI3K inhibitor LY294002 can inhibit the invasion phenotype of AGS and HGC-27 cell; scale bar, 50 μm. (C) LY294002 significantly inhibited the phosphorylation level of AKT, but the expression level of UFM1 did not change significantly. The phosphorylation level of AKT was significantly increased after knocking down UFM1. [file 13046_2019_1416_MOESM5_ESM.tif]

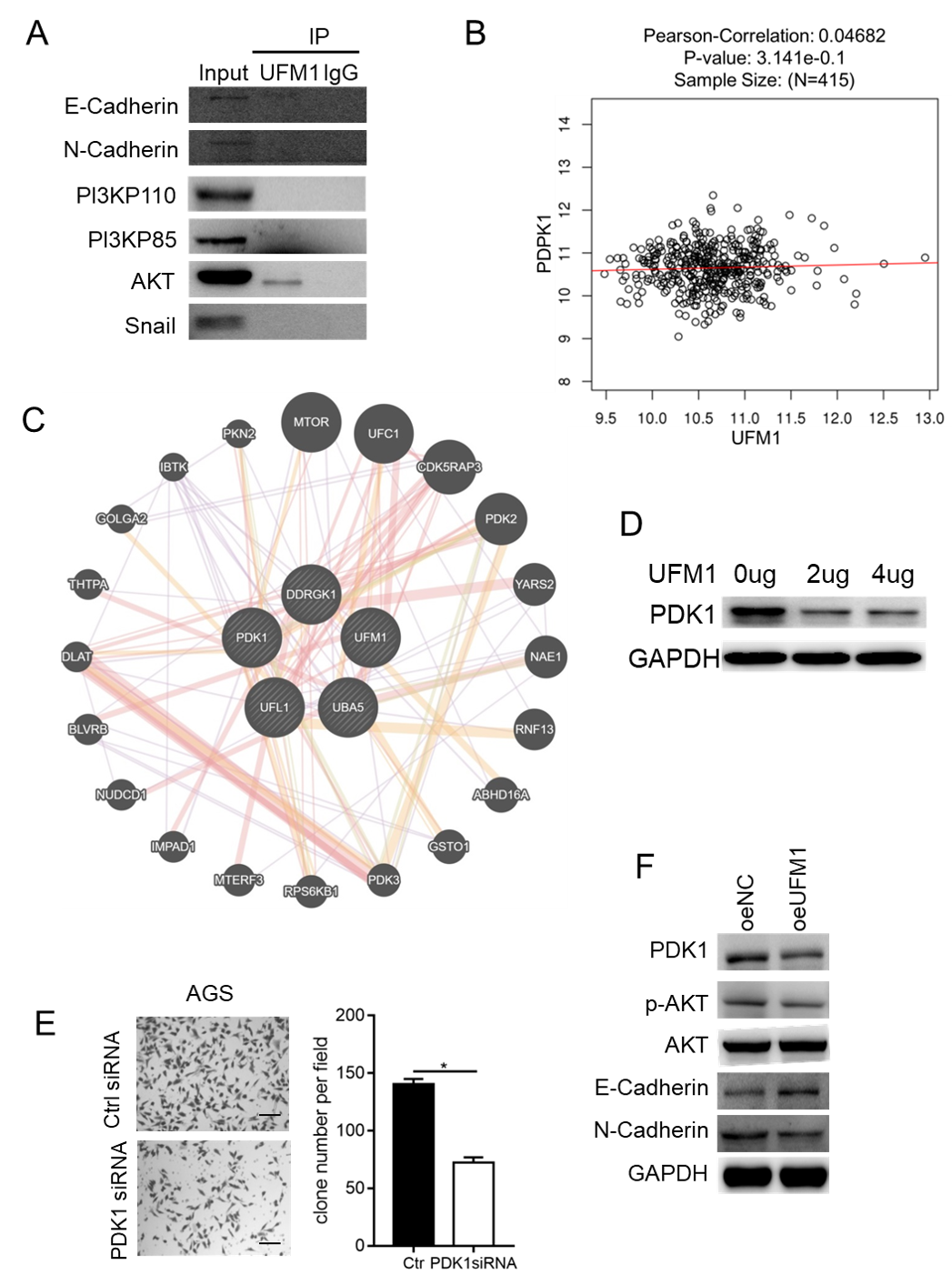

Supplement: Supplementary file 6 — Additional file 6: Figure S4. (A) The lysates of AGS cells were applied to immunoprecipitation using UFM1 antibody. The immunoprecipitates were examined to blot PI3K subunits p85 and p110, AKT, EMT-related proteins E-cadherin, N-cadherin and Snail. (B) The relationship of UFM1 and PDK1 in mRNA by Linkedomics browser. There was no obvious correlation between them (P = 0.314). (C) UFM1 modification system could interacts with PDK1 by the GeneMANIA browser. (D) AGS cells were transfected as indicated then applied to western blot. (E) PDK1 siRNA significant reduce AGS cell invasiveness. The data are presented as the mean ± SD; scale bar, 50 μm (*P < 0.05). [file 13046_2019_1416_MOESM6_ESM.tif]

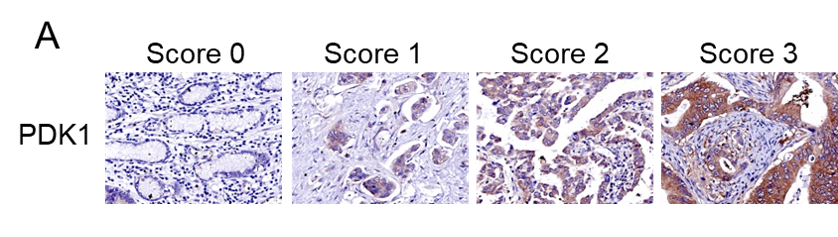

Supplement: Supplementary file 7 — Additional file 7: Figure S5. (A) Immunohistochemical staining of PDK1 expression in gastric cancer tissue and the criteria for immunohistochemistry scores following the intensity of positive signals, magnification, × 100. [file 13046_2019_1416_MOESM7_ESM.tif]
